# Falls and correlations among community-dwelling older adults: A Cross-sectional study in Jeddah, Saudi Arabia

**DOI:** 10.12669/pjms.39.1.6993

**Published:** 2023

**Authors:** Sultan H. Alamri, Ranya A. Ghamri, Wejdan H. Alshehri, Reema S. Alhuthayli, Nouf M. Alamoudi, Ragheed D. Alnufaei, Walid A. Alkeridy

**Affiliations:** 1 Sultan H. Alamri, Department of Family Medicine, Faculty of Medicine, King Abdulaziz University Hospital, Jeddah, Saudi Arabia Saudi Geriatrics Society, Saudi Commission for Health Specialties, Riyadh, Saudi Arabia; 2 Ranya A. Ghamri, Department of Family Medicine, Faculty of Medicine, King Abdulaziz University Hospital, Jeddah, Saudi Arabia; 3 Wejdan H. Alshehri, Department of Family Medicine, Faculty of Medicine, King Abdulaziz University Hospital, Jeddah, Saudi Arabia; 4 Reema S. Alhuthayli, Department of Family Medicine, Faculty of Medicine, King Abdulaziz University Hospital, Jeddah, Saudi Arabia; 5 Nouf M. Alamoudi, Department of Family Medicine, Faculty of Medicine, King Abdulaziz University Hospital, Jeddah, Saudi Arabia; 6 Ragheed D. Alnufaei, Department of Family Medicine, Faculty of Medicine, King Abdulaziz University Hospital, Jeddah, Saudi Arabia; 7 Walid A. Alkeridy, Dept. of Medicine, King Saud University, Riyadh, Saudi Arabia. Department of Medicine, Geriatric Division, University of British Columbia, Vancouver, Canada. Ministry of Health, Directorate of Home Health Care, Riyadh, Saudi Arabia. Saudi Geriatrics Society, Saudi Commission for Health Specialties, Riyadh, Saudi Arabia

**Keywords:** prevalence, risk, falls, injuries, older adults, community-dwelling, Jeddah

## Abstract

**Objectives:**

Falls are one of the major health issues faced by older adults, and they can result in physical harm, eventual loss of independence, and even death. Herein, we investigated the prevalence, alongside the main risk factors and resulting injuries, of falls among older adults.

**Methods:**

We employed a descriptive cross-sectional approach. Data were collected between February and July 2021 from 403 older adults aged 60 years or above via an online self-reported questionnaire. Basic activities of daily living (BADLs) and instrumental activities of daily living (IADLs) were also recorded.

**Results:**

The prevalence of falls among community-dwelling older adults was 47.4%. Among those who had experienced a fall, 36.2% incurred injuries, 25.3% had fractures, and 23.1% required walking aids. Age between 95-104 years, female sex, participants on anti-hypertensive medications, history of hip or knee replacement surgery, and presence of a caregiver, were significantly more likely to have had a previous history of falls (p < 0.05). Furthermore, having a previous history of stroke, osteoporosis, lower limb weakness, dizziness, using wheelchairs as walking aids, and living with the fear of stumbling or slipping were significantly associated with history of previous falls (p < 0.05).

**Conclusions:**

The prevalence of falls is high among community-dwelling older adults in Jeddah. Physicians should identify older adults with higher falling risk and provide them with appropriate interventions. Public health strategies could significantly reduce falls and fall-related injuries in older adults.

**List of Abbreviations:**
KSA: Kingdom of Saudi Arabia
BADLs: Basic activities of daily living
IADLs: Instrumental activities of daily living
SPSS: Statistical Package for the Social Sciences
χ2: Chi-squared test

## INTRODUCTION

Falls are one of the most serious health concerns faced by older adults worldwide. A fall is defined as an unintentional incident that results in an entity coming to rest on the ground or floor or other lower-level surface.^1^ According to a systematic review and meta-analysis published in 2019, falls are most prevalent (34–57.7%) among older adults.^2^ A study conducted in Riyadh city found that 49.9% of older adults in Saudi Arabia experienced one or more falls in a year.^3^

Multiple risk factors have been found to increase the risk of falls among older adults. These factors include chronic conditions such as cardiac disease and diabetes.^4^ The use of medication for chronic diseases and polypharmacy have also been reported to increase the risk of falls.^5^ Additionally, socioeconomic factors, such as low education level, 3,6 occupational status, unemployment, and low income, might also increase the predisposition to falls.^7^ It has also been shown that stress and using a walking aid are linked to higher chances of falling.^3^ Previous studies have shown that age is strongly associated with falls,^8^,^9^ suggesting that the prevalence of falls progressively increases with age.

Falls could also be linked to a variety of environmental hazards such as inappropriate styles of steps, poor lighting, clutter, slippery floors, and movable mats.^4^ The majority of falls (72.8%) have been reported to occur indoors.^8^ Overall, the prevalence of falls rises as the number of risk factors increases from 27%, among people with no risk factors or only one risk factor, to 78%, among those with several risk factors.^3^,^7^,^10^ Each year, emergency departments treat millions of people aged ≥ 65 for falls.^11^ Approximately 20% of the falls lead to serious injuries (such as fractures or head injuries), which may result in loss of functional ability, restriction in movement, loss of autonomy, or death.^4^,^12^

The financial burden of falls on health care and social systems is significant.^13^ Understanding the cause of falls could help establish effective prevention strategies for reducing the number of falls in older adults.^14^ Interventions such as education, exercise, environmental changes, and prescription review can effectively reduce the frequency of falls in high-risk communities.^15^

Multiple studies have evaluated the prevalence and associated risk factors of falls among older adults. However, to our knowledge, no such studies have been carried out in Jeddah, Saudi Arabia. Therefore, the objectives of this study were 1) to determine the prevalence of falls among community-dwelling older adults aged ≥ 60 years in Jeddah and 2) to identify the main factors associated with falls.

## METHODS

This cross-sectional study was conducted in Jeddah, Saudi Arabia, between February and July 2021. Older adults aged 60 years or above were surveyed for this study. A data collection Form was developed as a tool for questioning the participants. This was a four-page questionnaire consisting of 43 questions grouped under three main sections: demographic questions (age, sex, education, marital status, employment, and income), health status-related questions (chronic conditions, number of medications taken by participants), and fall-related questions (number of falls and related questions).

The Katz Index was used to evaluate independence in the Basic Activities of Daily Living (BADL). It is a reliable tool for evaluating the functional status of older adults.^16^ The index evaluates six primary functions: bathing, dressing, toileting, transferring, continence, and feeding. Independence to perform each function is scored with a yes/no answer. A score of six reflects full function, four reflects moderate functional impairment, and ≤ two indicates the presence of severe functional impairment.^17^

The Lawton Scale was used to evaluate independence in Instrumental Activities of Daily Living (IADLs). It is a standardized, validated tool used to determine the ability of older adults to care for themselves.^13^ This scale evaluates eight primary functions: (preparing one’s meals, using the telephone, going shopping, taking medications, performing light housework, doing laundry, transportation, and managing finances).^18^ Independence to perform each function is scored with a yes/no answer. The scores range from 0 (low function, dependent) to 8 (high function, independent).^19^

Participants’ data were collected using a standardized anonymous questionnaire, which was administered online in an electronic form to older adults living in Jeddah city. The sample size was calculated using the Roasoft online calculator. The calculation was based on a 50% response distribution, 5% margin of error and 95% confidence interval. The calculated sample size was 384 patients. Data were analysed using Statistical Packages for the Social Sciences (SPSS) version 25 software.

Qualitative data are expressed as frequencies or percentages, and the Chi-squared test (χ2) was used to analyze the relationship between variables. Quantitative data are expressed as mean with standard deviation (mean ± SD), and the Mann-Whitney test was used to analyze non-parametric variables. Binary logistic regression analysis was used to determine the independent predictors (risk factors) of falls among participants (odds ratio with 95% confidence intervals [CI]). A p-value of < 0.05 was considered statistically significant.

### Ethical approval and consent to participate:

The study was conducted in accordance with all local regulations and followed the principles of the Helsinki declaration. Informed consent for data collection was sought from participants before they answered the questions. Ethical approval was obtained from the Research Ethics Committee of King Abdulaziz University (Ref. No.: 51-21).

## RESULTS

A total of 403 participants completed our questionnaire. The greatest proportion of the participants aged 60–64 years (48.6%), were female (67.2%), and had a Saudi nationality (94.5%). Table-I Among our participants, 60.5% had < 5 children, 61.8% were married, 33.3% were illiterate, 23.1% had a household income in the range SAR10000–19999, and 33.3% were from the north Jeddah region.

**Table-I:** Distribution of participants according to their characteristics (No. 403).

Variable	No. (%)
***Age***	
60–64	196 (48.6)
65–74	110 (27.3)
75–84	58 (14.4)
85–94	35 (8.7)
95–104	3 (0.7)
^3^105	1 (0.2)
***Gender***	
Female	271 (67.2)
Male	132 (32.8)
***Nationality***	
Non-Saudi	22 (5.5)
Saudi	381 (94.5)
***Number of daughters/sons***	
<5	244 (60.5)
5–9	89 (22.1)
≥10	15 (3.7)
None	55 (13.6)
***Marital Status***	
Divorced	23(5.7)
Married	249 (61.8)
Single, never married	6 (1.5)
Widowed	125 (31)
***Educational level***	
High school	44 (10.9)
Bachelor	88 (21.8)
Diploma	26 (6.5)
Elementary school	60 (14.9)
Illiterate	134 (33.3)
Master’s degree or PhD	28 (6.9)
Middle school	23 (5.7)
***Household Income***	
10000–19999 SR	93(23.1)
5000–9999 SR	86 (21.3)
Less than 5000 SR	80 (19.9)
More than 19999 SR	45 (11.2)
No monthly income	52 (12.9)
Not mentioned	47 (11.7)
***Jeddah region***	
Centre	81(20.1)
East	74 (18.4)
North	134 (33.3)
Jeddah-Rural	47 (11.7)
South	56 (13.9)
West	11 (2.7)

Around 7.2% of the participants smoked cigarettes or shisha, 53.1% took between 1–4 drugs daily, and the most common medications taken were vitamin D (65.5%), anti-hypertension (HTN) (52.4%), and anti-diabetic medications (51.1%). About one-third (30.3%) of the participants had past surgical histories: mostly cataract surgery (40.2%), followed by orthopaedic surgery (17.6%) and heart procedures (11.4%). About 45% of the participants had a caregiver, and 82.4% owned their own residence. Most participants (66.7%) lived on a high floor, 38% had an elevator, and 40.4% were overweight with a mean body mass index (BMI) of 29.46 ± 6.45 kg/m^2^.

In terms of functional status, more than 70% of the participants were able to dress and feed themselves, count, use the toilet independently, bathe, transfer, make telephone calls, and take their medication by themselves. Only 27% participated in exercises regularly; among them, 23.1% were able to walk, 15.1% participated in daily exercises for ≥ 30 minutes, and 8.4% exercised ≥ 5 times weekly. The most prevalent chronic diseases included poor vision (53.8%), osteoarthritis (51.9%), back pain (51.6%), HTN (50.6%), and diabetes mellitus (50.4%). Most participants (64.5%) did not use walking aids, and 20.8% used walking sticks.

**Fig.1:**
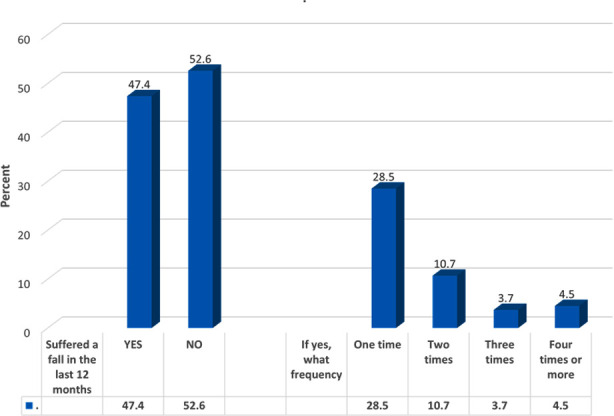
Percentage distribution of the participants according to previous falls in the last 12 months and their frequency.

In regard to falls, 47.4% of the participants had a previous history of falls; 28.5% of them had fallen only once (Fig.1). As illustrated Table-II , around 17.6% of the participants, reported occurrence of falls in the afternoon (12:00–5:59 p.m.), 14.1% had fallen on their side, 22.3% had fallen because the ground was slippery, 18.4% were able to get up by themselves, and 8.4% required hospital admission (for an overnight stay or longer). Only 23.1% needed walking aids after the incident. A total of 37.2% had fallen inside a house, most often in the bathroom (16.6%). Among those who had fallen in a public place, 3% fell in the street. After falling, 36.2% of the participants incurred injuries, 25.3% had fractures (most often in the arm), while 29.3% had bruises. Among the participants, 31.8% lived with fear of falling and 79.9% reported stumbling or slipping.

**Table-II:** Distribution of participants with a history of falls according to circumstances related to the fall and injury afterwards

Variable	No. (%)
***What time of a day had you fallen?***	
Afternoon (12:00 p.m.–5:59 p.m.)	71 (17.6)
Morning (6:00 a.m.–11:59 a.m.)	34 (8.4)
Morning (unspecific)	17 (4.2)
Night (12:00 a.m.–5:59 a.m.)	11 (2.7)
Night (6:00 p.m.–11:59 p.m.)	46 (11.4)
Night (unspecific)	12 (3)
***How did you fall?***	
Backward	61 (15.1)
Forward	41 (10.2)
On your knees	21 (5.2)
On your side	57 (14.1)
Other	11 (2.7)
***What was the cause of your fall?***	
Carpets	10 (2.5)
Dizzy/ lightheaded	25 (6.2)
Front doorstep	19 (4.7)
Light	9 (2.2)
Other	30 (7.4)
Slippery ground	90 (22.3)
Stairs	8 (2)
***Were you able to get up by yourself?***	
No	117 (29)
Yes	74 (18.4)
***Were you admitted to the hospital for an overnight or longer?***	
No	94 (23.3)
Yes	34 (8.4)
***After falling, did you need walking aids?***	
No	98 (24.3)
Yes	93 (23.1)
***Where did the fall occur?***	
Inside your home	150 (37.2)
Other	10 (2.5)
Public Place	25 (6.2)
Someone’s home	6 (1.5)
***If inside your home or someone else’s home; where exactly?*** (No.:150) ***else’s home; where exactly?***	
Bathroom	67 (16.6)
Bedroom	19 (4.7)
Front doorstep	11 (2.7)
Kitchen	16 (4)
Other	20 (5)
Stairs	17 (4.2)
***If a fall occurred in a public space; where exactly?*** (No.:25)	
Hospital	1 (0.2)
Mall	2 (0.5)
Mosque	1 (0.2)
Other	9 (2.2)
Street	12 (3)
***For those who suffered injury after falling*** (No.: 146)	
***After falling, were there any injuries?***	
No	45 (11.2)
Yes	146 (36.2)
***If yes, what was the type of injury:***	
Fracture	
No	102 (25.3)
Yes	44 (10.9)
***If the injury was a fracture, where exactly?***	
Arm	15 (3.7)
Hip	13 (3.2)
Leg	12 (3)
Other	28 (6.9)
Rib	4 (1)
Spine	6 (1)
Hairline fracture	40 (9.9)
Bruising	118 (29.3)
Laceration	39 (9.7)
loss of consciousness	30 (7.4)
Disc prolapse	30 (7.4)
***Are you afraid of falling?***	
Always	128 (31.8)
Never	50 (12.4)
Sometimes	225 (55.8)
***Do you stumble/slip?***	
Always	38 (9.4)
Never	81 (20.1)
Sometimes	284 (70.5)

The female sex, being 95−104 years old, having a middle educational level, and having a household income less than SAR5000 were all associated with a significantly higher risk of having a previous fall (p < 0.05). Table-III Conversely, a non-significant association was found between a history of previous fall(s) and participants’ nationality, number of children, or marital status (p > 0.05).

**Table-III:** Relationship between fall history and participants’ characteristics.

Variable	History of fall		χ2	p value

	Present No. (%)	Absent No. (%)		
***Age (years)***			23.04	<0.001
60–64	1 (100)	0 (0.0)		
65–74	74 (37.8)	122 (62.2)		
75–84	52 (47.3)	58 (52.7)		
85–94	40 (69)	18 (31)		
95–104	22 (62.9)	13 (37.1)		
≥105	2 (66.7)	1 (33.3)		
***Gender***			5.04	0.025
Female	139 (51.3)	132 (48.7)		
Male	52 (39.4)	80 (60.6)		
***Nationality***			2.26	0.132
Non-Saudi	7 (31.8)	15 (68.2)		
Saudi	184 (48.3)	197 (51.7)		
***Number of daughters/sons***			0.45	0.928
<5	117 (48)	127 (52)		
5–9	40 944.9)	49 (55.1)		
≥10	8 (53.3)	7 (46.7)		
None	28 (47.3)	29 (52.7)		
***Marital Status***			6.77	0.079
Divorced	11 (47.8)	12 (52.2)		
Married	106 (42.6)	143 (57.4)		
Single, never married	3 (50)	3 (50)		
Widowed	71 (58.8)	54 (43.2)		
***Educational level***			19.53	0.003
High school	16 (38.4)	28 (63.6)		
Bachelor Diploma	28 (31.8) 11 (42.3)	60 (88.2) 15 (57.7)		
Elementary school	35 (58.3)	25 (41.7)		
Illiterate	76 (56.7)	58 (43.3)		
Master’s degree or PhD	12 (42.9)	16 (57.1)		
Middle school	13 (58.5)	10 (43.5)		
***Household Income***			18.4	0.002
10000–19999 SAR	33 (35.5)	60 (64.5)		
5000–9999 SAR	32 (37.2)	54 (62.8)		
Less than 5000 SAR	49 (61.3)	31 (38.8)		
More than 19999 SAR	21 (46.7)	24 (53.3)		
No monthly income	29 (55.8)	23 (44.2)		
Not mentioned	27 (57.4)	20 (42.6)		

Participants on anti-hypertensive medications, who have a surgical history of hip or knee replacement surgery, who have a caregiver, and who have a rented residence were significantly more likely to have had a previous history of falls (p < 0.05).Table-IV A non-significant relationship was found between having a previous history of falls and participants’ smoking status, intake of medications apart from anti-HTN medication, surgical history other than hip or knee replacement surgery, being bedridden, type of floor, or BMI categories (p > 0.05).

**Table-IV:** Relationship between fall history and participants’ smoking status, medication, surgical history, type of residence and floor, and BMI categories.

Variable	History of fall		χ2	p value

	Present No. (%)	Absent No. (%)		
***Do you smoke (Cigarettes or Shisha)***				
No	190 (48.1)	194 (51.9)	1.12	0.289
Yes	11 (37.9)	18 (62.1)		
***Do you take any medications? How many?***				
1 to 4 drugs	99 (46.3)	115 (53.7)	5.45	0.065
5 or more drugs	71 (54.2)	60 (45.8)		
Do not use	21 (36.2)	37 (63.6)		
***Medications***				
Vitamin D	133 (50.4)	131 (49.6)	2.73	0.098
Anticoagulants/Antiplatelets	61 (52.1)	56 (47.9)	1.48	0.223
Antiepileptic drugs	8 (57.1)	6 (42.9)	0.55	0.457
Antidepressants	23 (62.2)	14 (37.8)	3.56	0.059
Sleep medications	38 (58.5)	27 (41.5)	3.8	0.051
Heart medications	43 (53.8)	37 (46.3)	1.61	0.204
Anti–HTN medications	110 (52.1)	101 (47.9)	3.98	0.046
Diabetes medications	106 (51.5)	100 (48.5)	2.78	0.095
***Do you have any past surgical history?***				
No	142 (50.5)	139 (49.5)	3.66	0.055
Yes	49 (40.2)	73 (59.8)		
***Type of surgical history:***				
Brain surgery	4 (44.4)	5 (55.6)	0.03	0.858
Urological surgery	15 (39.5)	23 (60.5)	1.05	0.304
Ear surgery e.g., middle ear surgery	8 (50)	8 (50)	0.04	0.831
Eye surgery, e.g., cataract surgery	83 (51.2)	79 (48.8)	1.6	0.206
Hip or knee replacement surgery	18 (72)	7 (28)	6.47	0.011
Orthopedic surgery	40 (46.3)	31 (43.7)	2.76	0.096
Heart procedure/surgery	42 (52.2)	22 (47.8)	0.47	0.49
[Other]	51 (50.5)	50 (49.5)	0.52	0.47
***Do you have a caregiver,*** e.g.,***nurse?***				
No	92 (41.3)	131 (58.7)	7.54	0.006
Yes	99 (55)	81 (45)		
***Type of residence***				
Owned	149 (44.9)	183 (55.1)	4.78	0.029
Rented	42 (59.2)	29 (40.8)		
***Type of floor?***				
High floor	121 (45)	148 (55)	1.89	0.169
Low floor	70 (52.2)	64 (47.8)		
***If high floor, Elevator vs. No elevator*** (No.:269)				
Elevator				
No elevator	69 (45.1)	84 (54.9)	1.89	0.388
	52 (44.8)	64 (55.2)		
***BMI categories***				
Underweight	3 (60)	2 (40)		
Normal weight	36 (45)	44 (55)	0.74	0.863
Overweight	80 (49.1)	83 (50.9)		
Obese	72 (46.5)	83 (53.5)		
***BMI (mean ± SD)***	29.23 ± 6.48	29.67 ± 6.44	0.56	0.574

In relation to function, participants who were able to bath, do shopping, do transportation, to cook food, to take medication, do housework, laundry, and accounting were significantly less likely to have history a previous fall (p < 0.05). Furthermore, having a previous history of stroke, osteoporosis, lower limb weakness, dizziness, using wheelchairs as walking aids, and living with the fear of stumbling or slipping were significantly associated with a history of previous falling (p < 0.05). However, a non-significant association (p > 0.05) was found between previous history of falls and other chronic diseases and the possibility to perform other tasks or practice physical exercises, irrespective of the duration, frequency, or type of exercise.

The binary logistic regression analysis was used to determine the independent predictors (risk factors) of falls among participants. It showed that age between 95–104 years, the female sex, recurrent stumbling or slipping, living in a rented residence, the presence of a caregiver, and the inability to use transportation devices were all independent predictors (risk factors) of fall among the participants (p < 0.05, CI: 95%).

## DISCUSSION

Falls are common among older adults and could lead to serious injuries, significantly impacting morbidity and mortality.^4^ The purpose of this study was to evaluate the prevalence of falls among older adults and determine the associated risk factors and injuries. Identifying risk factors is critical for preventing falls and reducing the likelihood of injury. It can also aid in preventing secondary morbidities, thereby improving the quality of life of older adults.^3^,^20^,^21^

The results of the present study showed that the prevalence of falls among older adults in Jeddah was 47.4%. Similar studies have shown that 49.9% and 42.4% of older adults in Saudi Arabia and Canada had a history of falls, respectively.^3^,^22^

Remarkably, a variation in prevalence rate was reported in multiple countries, 34%, 34%, 32%, 28%, 27.6%, 19%, 16%, 11%, and 4% in Canada, Qatar, USA, England, Brazil, Hong Kong, Japan, China, and Malaysia, respectively.^7^,^11^,^20^–^23^ A difference in the incidence and prevalence of chronic illnesses between countries, the use of multiple drugs that may interact with patients’ quality of life, and the difference in environmental factors are all possible explanations for these differences.

In our study, 51.3% of female participants had a history of falls compared to 39.4% of older males. This finding is consistent with that of a study in Unaizah, Saudi Arabia (KSA), which showed a prevalence of 34.5% and 28.5% among older females and older males, respectively. The higher prevalence in females could be due to lack of education and low-income level, both of which have been shown to have a significant correlation with a history of falling.^24^

In this study, chronic diseases were found to be a risk factor for falls, as demonstrated in previous studies.^24^ The most common chronic health condition reported by participants in this study was poor vision. Surprisingly, only 48.4% of participants with poor vision had experienced a falling event. However, in concordance with the findings in previous studies, no significant association was found between these two parameters.^3^,^23^ The most common cause of falls was dizziness (58.3%), which increases the risk of falls due to balance problems associated with aging.^25^ Balance disorders could have a variety of causes (such as unstable gait, ear infections, visual impairments, arthritis, and confusion caused by psychoactive medications).^25^

Not surprisingly, participants with lower-limb weaknesses in this study had a significantly higher risk of falling. These results are in line with previous studies.^1^ Osteoporosis was also found to be a significant risk factor for falls, in concordance with previous studies.^23^ In our study, approximately 36.2% sustained post-fall injuries. The most common injuries were bruising, hairline fractures, laceration, and arm and hip fractures. This is consistent with previous reports.^25^

The present study also revealed a significant association between a hip or knee replacement surgery and risk of falls. These results concurred with those observed in earlier studies.^1^ One unanticipated finding was that older adults with a caregiver had a significantly high risk of falls. This was also evident in a study by Almegbel et al., 2018.^3^ However, this might be due to the retrospective nature of the study where some patients might have been assigned caregivers after they had experienced the fall. This could explain why most of the participants who had a previous history of fall had caregivers, and will not necessarily imply that having a caregiver is associated with an increase in the risk of falling.

Moreover, older age was found to be a factor associated with falls in our study, as reported in several previous studies carried out in developed countries.^26^ In a Japanese study, and after age-standardization, the proportion of falls in one year for both native Japanese and Japanese Americans living in Hawaii was approximately half that in published Caucasian studies.^3^,^27^ This suggests that the disparity in the prevalence of falls may be due to genetic rather than environmental factors. These results contradict a study conducted in Riyadh, Saudi Arabia, in which no significant association was found between aging and falls among older adults.

Additionally, the present study revealed a high prevalence of falls among older adults with a low educational level. This finding is also consistent with the results of other studies.^3^ Polypharmacy was found to be a non-significant risk factor for falls among the older population in this study. However, other studies found a significant relationship between falls and polypharmacy in older adults.^3^ These differences may be related to the multiple comorbidities for which these medications were prescribed rather than the medications themselves.

It was also found that participants who lived in a rented home had a considerably greater risk of falling (p<0.05). This finding matches the results of a study in Riyadh, KSA.^3^ Furthermore, our study showed that the majority of falls inside homes (37.2%) occurred mostly in the bathroom (16.6%). This is similar to results from a Malaysian study by Yeong et al.^23^ The same finding was observed in a previous Saudi study where indoor falls were most common in frequently used areas, including the kitchen, bedroom, and living room.^3^ Our findings also concurred with previous studies that showed that using a walking aid was associated with a higher risk of falls among older adults.^9^

### Limitations:

First, we used a one-year recall period, which is susceptible to inducing recall bias. Second, other associated factors, such as postural hypotension, cognitive impairment, and malnutrition, were not included in our survey; it may be useful to evaluate these other factors in future studies.

## CONCLUSIONS

Falls were highly prevalent among community-dwelling older adults in Jeddah. Advanced age, female sex, use of anti-hypertensive medications, history of hip or knee replacement surgery, having a caregiver, previous history of stroke, osteoporosis, lower limb weakness, dizziness, using wheelchairs as walking aids, and living with the fear of stumbling or slipping were significantly associated with falls. These findings corroborate the findings of a previous study conducted in Riyadh.^4^ Physicians should identify older adults most at risk of falling and provide appropriate interventions. Public health strategies can significantly reduce falls and fall-related injuries in older adults.

### Authors Contributions

**SHA, WHA, and RSA** conceptualized and designed the study, developed the questionnaire, and drafted the manuscript. They also assisted in data collection and shared in statistical design and analysis.

**NMA and RDA** participated in the study planning and assisted in data collection and entry.

**RAG and WAA** helped with data interpretation and manuscript editing.

**SHA, RAG and WAA** were responsible for the overall accuracy of this project.

**All authors** contributed to the final revision of the manuscript and approved it for publication.
